# Development of the intelligent knee osteoarthritis lifestyle app: a person-based approach

**DOI:** 10.1186/s12891-024-07313-4

**Published:** 2024-03-02

**Authors:** Richard D. M. Stevenson, Enhad A. Chowdhury, Victor B. Inza, Max J. Western, Nicola E. Walsh, Simon L. Jones, James L. J. Bilzon

**Affiliations:** 1 Department for Health, The University of Bath, Bath, BA2 7AY UK; 2 Centre for Sport, Exercise and Osteoarthritis Research Versus Arthritis, University of Bath, Bath, UK; 3 Department of Computer Science, The University of Bath, Bath, BA2 7AY UK; 4 Visual Computing Centre, King Abdullah University of Science and Technology, Thuwal, Saudi Arabia; 5 Faculty of Health and Applied Science, University of the West of England, Bristol, UK

**Keywords:** Knee osteoarthritis, Self-management, Physical activity, Mobile application, Digital health

## Abstract

**Background:**

Knee osteoarthritis is one of the most prevalent long term health conditions globally. Exercise and physical activity are now widely recognised to significantly reduce joint pain, improve physical function and quality of life in patients with knee osteoarthritis. However, prescribed exercise without regular contact with a healthcare professional often results in lower adherence and poorer health outcomes. Digital mobile health (mHealth) technologies offer great potential to support people with long-term conditions such as knee osteoarthritis more efficiently and effectively and with relatively lower cost than existing interventions. However, there are currently very few mHealth interventions for the self-management of knee osteoarthritis. The aim of the present study was to describe the development process of a mHealth app to extend the support for physical activity and musculoskeletal health beyond short-term, structured rehabilitation through self-management, personalised physical activity, education, and social support.

**Methods:**

The development of the intelligent knee osteoarthritis lifestyle application intervention involved an iterative and interconnected process comprising intervention ‘planning’ and ‘optimisation’ informed by the person-based approach framework for the development of digital health interventions. The planning phase involved a literature review and collection of qualitative data obtained from focus groups with individuals with knee osteoarthritis (*n* = 26) and interviews with relevant physiotherapists (*n* = 5) to generate ‘guiding principles’ for the intervention. The optimisation phase involved usability testing (*n* = 7) and qualitative ‘think aloud’ sessions (*n* = 6) with potential beneficiaries to refine the development of the intervention.

**Results:**

Key themes that emerged from the qualitative data included the need for educational material, modifying activities to suit individual abilities and preferences as well as the inclusion of key features such as rehabilitation exercises. Following a user-trial further changes were made to improve the usability of the application.

**Conclusions:**

Using a systematic person-based, development approach, we have developed the intelligent knee osteoarthritis lifestyle application to help people maintain physical activity behaviour. The app extends the support for physical activity and musculoskeletal health beyond short-term, structured rehabilitation through personalised physical activity guidance, education, and social support.

**Supplementary Information:**

The online version contains supplementary material available at 10.1186/s12891-024-07313-4.

## Background

It is estimated that more than 240 million people globally are affected by osteoarthritis (OA) [1] which limits people’s daily activities [2] leading to physical disability and impairing quality of life [3]. The condition can affect almost any joint, but OA of the knee and hip are among the most common and debilitating conditions [1, 4]. People with knee osteoarthritis (KOA) suffer with pain, stiffness and joint dysfunction and consequently are more sedentary and have more comorbidities than those without [3, 5]. Because of this, exercise and physical activity (PA) is widely recommended and has been shown to significantly reduce pain, improve physical function and quality of life in those with KOA [5–8].

Unfortunately, there are numerous barriers to PA amongst people with KOA including pain and functional limitations, a lack of knowledge regarding the effects of exercise and a lack of social/professional support [9]. Whilst prescribed exercise can negate some barriers, the beneficial effects of exercise interventions decline after individuals cease activity [10], which is problematic, as exercise prescription without regular contact with a healthcare professional often results in decreasing adherence and poorer health outcomes [11, 12]. In response to this problem, a range of interventions have been developed to maintain exercise adherence including face to face booster sessions, telephone sessions, community walking and group rehabilitation programmes [13–15].

A number of digital interventions using electronic health (eHealth) and mobile health (mHealth) technologies have also been developed for KOA [16, 17] which offer great potential to deliver interventions more efficiently, effectively and with relatively lower costs compared to more traditional face-to-face care [18, 19]. However, it is recognised that to enhance the likelihood of longer-term success, these technologies should be based on empirical behavioural theory [20] and adopt a person-based approach to development to ensure they meet the needs of target users [21, 22].

To date, the majority of mHealth apps for KOA have tended to focus on areas such as mobile assessment (related symptoms and pain), measurement (for joint range) as well as motion monitoring tools (gait and exercises) [23] with only a few focusing on specific elements of lifestyle management [24, 25]. To our knowledge, there are no mHealth apps that combine personalised PA plans, education, and social support in a single application. The aim of the present study was to develop and optimise a novel digital app-based intervention for the self-management of KOA with target users. Accordingly, this paper describes the development process of a theory-, evidence- and person-based mHealth app to extend the support for PA and musculoskeletal health beyond short-term, structured rehabilitation through personalised PA, self-management, education, and social support.

## Methods

The development of the intelligent Knee Osteo-Arthritis Lifestyle App (iKOALA) intervention involved an interconnected 2-stage process comprising intervention ‘planning’ and ‘optimisation’ which was informed by the person-based approach (PBA) framework for the development of digital health interventions [22]. Briefly, the PBA is an intervention development framework that places the intended target user at the centre of the research so that their life context and any barriers to engagement with the app or the behaviours it endorses are recognised and addressed in the design of the intervention. The objective of the PBA is to maximise the usability, motivation, and ultimately benefit of the intervention.

**Fig. 1:**
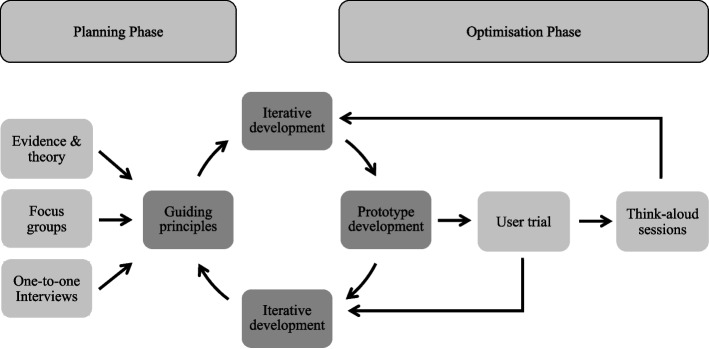
Overview of the iKOALA intervention development process based on the PBA

In the present study the ‘planning’ phase of the PBA involved collecting data from a range of sources including a literature review and primary qualitative data obtained from focus groups with individuals with KOA and interviews with physiotherapists to generate ‘guiding principles’ for the intervention (Fig. 1). The ‘optimisation’ phase involved usability testing and qualitative ‘think aloud’ sessions with potential beneficiaries to refine the development of the intervention. In reporting this user-centred development process, we also followed the Guidance for Reporting Involvement of Patients and the Public (GRIPP2, see Supplementary file) and associated checklist [26]. Ethical approval for the study was provided by the Department for Health Research Ethics Committee (REACH) at the University of Bath (Ref: EP 18/19 080).

### Intervention planning

#### Evidence and theory

A scoping review was undertaken to establish the evidence and theory relating to (i) the efficacy and safe implementation of exercise for individuals with KOA and (ii) the current digital interventions available for KOA management in the United Kingdom, to determine any unmet needs in these interventions. A literature search was undertaken using PubMed, Medline and EMBASE up until the 31st of December 2018. Sources consulted included original research articles, reviews, position stands and meta-analyses in the academic literature. The key search terms used were “knee osteoarthritis”, “physical activity” and “management” and “osteoarthritis” and “electronic” and “management” from 2005 to 2018. Only human studies written in English were included in the search. The articles included in this review aimed to identify the key literature on the subject, however this was not a full systematic review.

The theoretical basis for the iKOALA intervention drew from Self-Determination Theory [27]. SDT propagates that conditions that support the needs for autonomy (i.e., the need to feel volitional control over one’s behaviour), competence (i.e., the need to feel mastery over tasks or behaviours) and relatedness (i.e., the need to feel connected to others) will lead to higher quality internalised forms of motivation, better engagement with health behaviours and improved wellbeing [28]. Recent evidence from SDT that informed the iKOALA guiding principles comes from the Motivation, Engagement and Thriving in User Experience (METUX) model that champions the importance to satisfy the basic needs at various spheres of user interaction, namely the decision to sign-up to an app, the set-up process, the interface and the behaviours the app promotes [29]. We also draw from the motivational behaviour change techniques that endorses strategies for supporting the basic needs [30], such as offering choice, provision of meaningful rationale and informational (rather than prescriptive) feedback for autonomy, facilitating positive social support, and using empathetic respective language for relatedness, and using graded-tasks, optimising challenge, and self-monitoring for competence.

A final guiding framework was the COM-B model, which stipulates that behaviour is a product of not only motivation (which we target using SDT) but also the induvial opportunity and capability to undertake the desired behaviours [31]. Accordingly, we recognise the importance of tailoring any advice and motivational support to the individual physical and psychological capabilities of the user by understanding limits to their pain and physical function and providing clear instructions on how to exercise. We also acknowledge the importance of ensuring recommendations of a digital solution account for the physical and social opportunities of users, for example by accounting for time, money and access issues, and the preferences towards social or individual activities.

#### Focus groups

Four focus group were conducted with individuals diagnosed with KOA to gain a deeper understanding of the impact of living with OA and the needs of those wishing to maintain safe and appropriate exercise and PA. Participants were recruited through the University of the Third Age (https://www.u3a.org.uk/) as well as through social media advertising. All trial participants were deemed eligible if they had a diagnosis of KOA which was not caused by an acute knee injury. Participants included individuals with varying degrees of OA from those who had not engaged in any formal treatment for KOA to those who had undergone knee replacement surgery. All participants provided written informed consent to participate in the study.

Focus groups, led by members of the research team were semi-structured in nature, using digital polling software (https://www.turningsolutions.com) along with pre-determined questions to stimulate group discussion. Focus groups 1a (*n* = 9) and 1b (*n* = 7) (led by MW and EC) were conducted at the University of Bath, England in November of 2018 with the aim of gaining a better understanding of the general attitudes to KOA, the approach to, and views of PA and self-management and the views on using digital technologies for KOA management. Focus groups 2a (*n* = 6) and 2b (*n* = 4) (led by EC and SJ) were conducted between July and November 2020 at the University of Bath with the aim of focusing on the features that should be included in a self-management app for KOA, including what activities should be incorporated, how the intervention could accommodate a range of physical capabilities, and how the intervention could support motivation to maintain PA. Focus groups were audio or video recorded with prior consent and subsequently transcribed verbatim.

#### Individual interviews

Individual qualitative interviews with physiotherapists were conducted at several locations in Bath, between February and April 2020. Individuals were recruited through social media advertising. All participants provided written informed consent to participate in the study. To be eligible to participate, individuals were required to be a practicing chartered physiotherapist with experience of treating individuals with KOA. Physiotherapists were from a range of backgrounds, including working within the National Health Service (NHS) as well as in private practice. Interviews were conducted by a member of the research team (EC or SJ) and were semi-structured in nature using a set of pre-determined questions. The interviewer explained to participants that we were interested in developing a smartphone app to support individuals with KOA to enhance and/or maintain PA.

The aim of these interviews was to gain a better understanding of how physiotherapists approached promoting, recommending, and adapting exercises and physical activities for patients with KOA. Through the interview we aimed to determine if there were any guiding principles, common approaches or decision making processes for the management of KOA. This included the physiotherapists being shown 4 broad categories of PA along with a number or exercises associated with those categories. These were: mind/body (yoga, tai chi, gardening), sports (tennis, lawn bowls, golf, walking football), dance (line dancing, Zumba, exergaming) and cardiovascular exercise (Nordic walking, jogging, rowing, cycling, swimming). Participants were asked to select an activity from each category and describe how they would determine if the activity was suitable for the patient whilst at the same time considering any physical (physical function, pain severity, current activity level, contraindications to exercise) and psychosocial (emotional wellbeing, social support, confidence to self-manage, access to resources, understanding of diagnosis/treatment) factors*.* Finally, participants were asked which features they would want to see in an app to support engagement in PA and what might motivate them to use such an app with patients. Individual interviews were audio or video recorded, with prior consent and transcribed verbatim.

#### Development of intervention guiding principles

The development of intervention guiding principles is a core component of the PBA [32]. These guiding principles seek to highlight solutions (features or design objectives) that the intervention should incorporate to overcome any challenges to engagement on the part of target users. In this study, the guiding principles were developed iteratively from the theoretical and empirical literature along with qualitative data generated from user focus groups and individual interviews with physiotherapists.

### Intervention optimisation

#### User trial

In January 2021, a 3-week user trial of the iKOALA intervention commenced with the aim of gaining feedback on the most up-to-date version of the iKOALA intervention. Participants were recruited from the previous focus groups where individuals indicated they would be interested in taking part in further research having given their consent to be recontacted. To be eligible participants were required to be aged 45 years or older, have experienced chronic knee pain for a period of three months or longer or been diagnosed radiographically or clinically with knee OA by a clinician, and not had knee pain primarily arising from an acute knee injury. Finally, participants must not have had any other condition, or a treatment related to another condition that prevented them from participation in PA.

Each participant was provided with a PA tracker (Fitbit Inspire HR, Fitbit, USA) and a smartphone (Samsung A10, Samsung, Korea) with version 10 operating system with both the Fitbit and iKOALA applications pre-installed on the device. Participants were asked to create accounts for the Fitbit and iKOALA app and to link the two through the settings function in iKOALA. Participants were then instructed to complete the iKOALA PA questionnaire which consisted of a series of questions with nominal answers relating to their current level of PA and PA preferences:

1. How would you describe your level of PA?
2. Have you experienced any falls or serious difficulties with your balance?
3. Would you like to perform PA by yourself or in a group, at home or away from home, indoors or outdoors?
4. Would you prefer to organise your own group activities or attend instructor led classes?
5. Would you be happy to perform activities that may involve some cost?
6. Would you be happy to buy your own equipment?
7. Would you be interested in gym based, water based, sport or dancing activities?

**Fig. 2:**
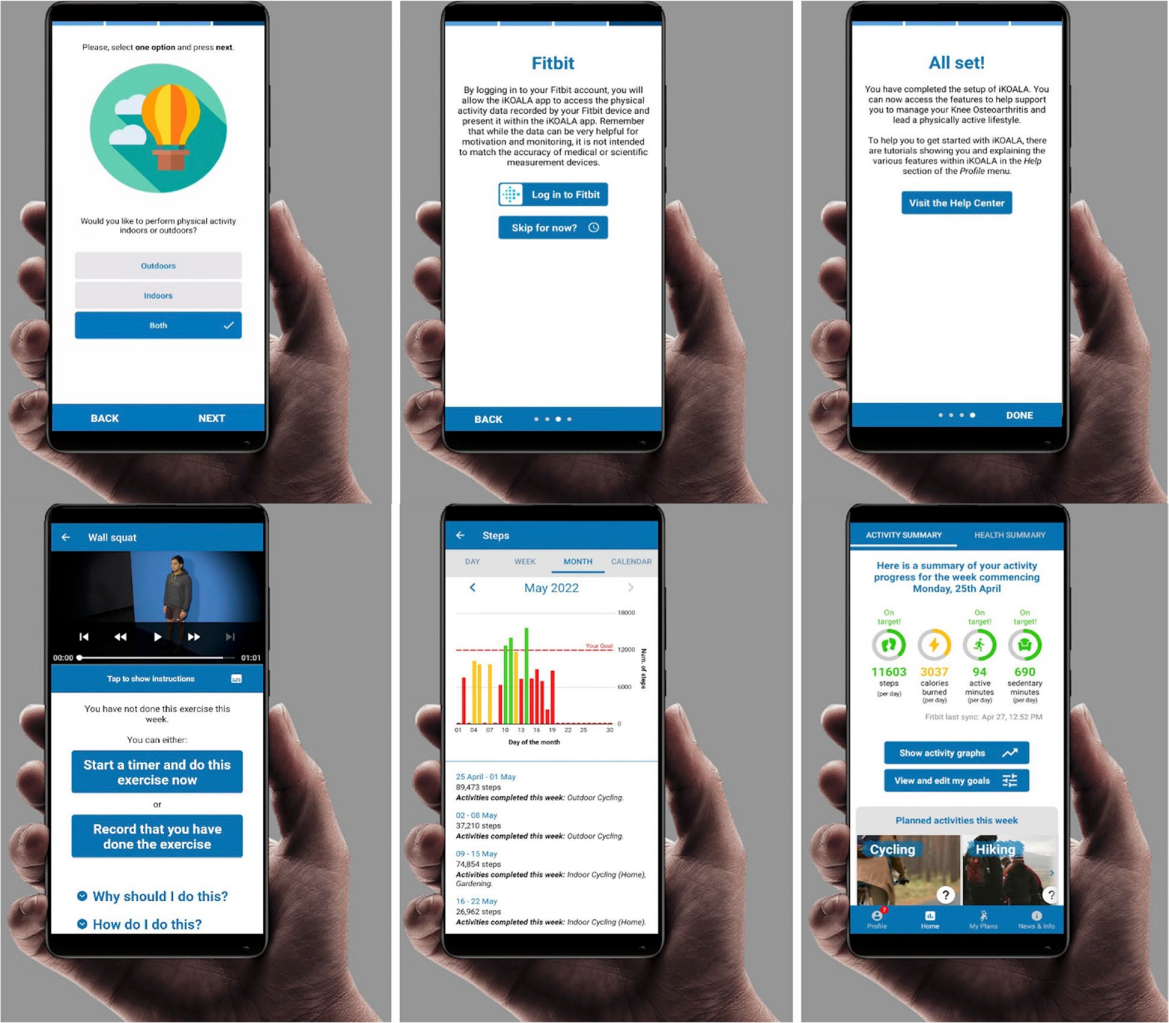
Screenshots of the iKOALA setup process (top) and of the features (bottom)

Based on the answer to question one (current levels of PA), users were assigned varying daily step goals from their answers; not active at all (8,000 steps), I do some activity (10,000 steps) and I am very active (12,000 steps). Participants were asked to wear their PA tracker during waking hours throughout the 3-week trial, which monitored daily activity in steps. This information was automatically displayed on the iKOALA app providing feedback on their PA goals. Based on the answers to questions two to seven (PA preferences), a list of suitable activities were presented to the individual on the iKOALA app. Users were required to select several of the recommended exercises adding them to their personalised PA plan to assist them in safely meeting their PA goals. Following this, participants were asked to familiarise themselves with the app and to log any PA sessions completed in the app. Participants were encouraged to use the iKOALA intervention to monitor their PA and symptoms to help them achieve their PA goals (Fig. 2).

At the end of each week participants were asked to complete a paper user diary indicating what features were used, what was liked about the app, what was disliked about the app, what issues (crashing, display issues, features not working etc.) were encountered (if any), and what thoughts or questions arose whilst using the app. At the end of weeks 1 and 2, participants were contacted by a researcher (EC) to ensure that there were no major issues with either the smartphone or Fitbit app or the iKOALA functionality. At the end of the 3 weeks, participants were asked to complete a 10-item system usability scale (SUS) Likert questionnaire to evaluate the subjective useableness of the intervention [33]. Finally, following completion of the user trial, participants were also invited to take part in an evaluation interview by video conference to gain their views on the iKOALA intervention. The interview contained a series of open ended questions (such as “can you tell me about anything you thought was particularly good about the app”). The user diaries and interviews were audio or video recorded, with prior consent and transcribed verbatim.

#### Think-aloud sessions

Six of the seven participants involved in the user trial gave their consent to be contacted and subsequently re-consented to take part in the qualitative think-aloud sessions. These were conducted via videoconference in May 2020. Think-aloud sessions were conducted by members of the research team (EC, SJ and VI) along with the individual participants one at a time. During each think-aloud session, participants were given access to a smartphone with the prototype iKOALA intervention on (containing the same pre-set fictitious data). Participants were asked to navigate in the app to complete a series of specific tasks and to answer several questions directed by the researcher (such as “can you tell me what activities are currently in the activity plan”?). The researcher used prompts where necessary. Throughout the session, participants were asked to ‘think out loud’ and to talk through the process of what they were thinking and doing during each task. In the final 10–15 min of the session, the researcher asked a series of summary questions to gain the views of participants about the app (such as “can you tell me anything you thought was particularly good about the app”? and “is there anything about how the app works in general or navigation that you would like to comment on”?). Think-aloud interviews were audio or video recorded, with prior consent and transcribed verbatim.

#### Analysis

Descriptive quantitative data are presented as mean, standard deviation (SD) and range unless otherwise stated. Qualitative data were transcribed and analysed thematically based on an inductive approach, guided by the process of Braun and Clarke (2006) that involves (1) becoming familiar with the data, (2) creating initial codes, (3) searching for themes, (4) reviewing the themes, (5) defining and naming the themes and finally (6) interpreting them within the context of the project. NVivo 7 (http://www.qrsinternational.com/) was used to organise and manage then qualitative data for the thematic analysis. All identifiable participant data was anonymised for confidentiality purposes.

Themes interpreted in the qualitative data from the planning phase (focus groups and physiotherapist interview) were presented along with participant quotes. Qualitative data from the optimisation phase (think-aloud interviews and user trial) were analysed using the ‘table of changes’ method (https://www.personbasedapproach.org/table_of_changes.html) of the PBA [32]. After (1) undertaking the think-aloud sessions and user trial feedback interviews, these were (2) transcribed verbatim. These transcriptions were then used to (3) draw out key positive and negative comments about specific elements or features of the intervention and these were (4) discussed with the intervention development team to identify the ways in which these problems may be solved. The (5) MoSCoW (Must do, Should do, Could do, Would like to do) criteria were then used to prioritise changes and (6) the agreed changes that were considered essential and achievable were subsequently implemented into the intervention. These results were presented summarising the feedback and associated changes made to the iKOALA intervention.

## Results

Table 1 presents the characteristics of participants that took part in the focus groups, qualitative interviews, user trial and the think-aloud sessions during the study.

**Table 1:** Participant characteristics in the various phases of the study

Phase	n	Characteristic	
		*Gender*	*Age (mean (SD), range*
Focus group 1a	9	5 male, 4 female	62 (± 7), 55–71
Focus group 1b	7	2 male, 5 female	60 (± 6), 51–67
Focus group 2a	6	2 male, 4 female	60 (± 10), 53–75
Focus group 2b	4	1 male, 3 female	63 (± 7), 56–71
		*Gender*	*Age (mean (SD), range*
Qualitative	5	2 male, 3 female	41 (± 11), (26–54)
Interviews with		*Years of practice*	*Years working with OA patients*
Physiotherapists		1 (1–5 years)	1 (1–5 years)
		2 (10–20 years)	2 (10–20 years)
		2 (20 + years)	2 (20 + years)
		*Gender*	*Age (mean (SD), range*
User trial	7	3 male, 4 female	63 (± 8), 49–71
		*Gender*	*Age (mean (SD), range*
Think-aloud session	6	3 male, 3 female	61 (± 9), 55–71

Unless otherwise stated, participants used were individuals with KOA and/or chronic knee pain

### Intervention planning

#### Evidence and theory

In relation to the effective and safe implementation of exercise for individuals with KOA, a total of eighty-four publications were identified from the databases of which thirty-nine were duplicates and forteen deemed unsuitable leaving thirty-one articles [3, 5, 6, 8, 10–12, 14, 15, 34–55]. For the KOA digital interventions, a total of forteen publications were identified from the databases of which six were duplicates and three deemed unsuitable leaving five articles [24, 44, 56–58].

In terms of evidence, our scoping review did not establish or promote clear superiority of a certain modality over another [5, 42, 53, 59]. The lack of focus on specific exercise modes also tallies with information from OA charities, where information emphasises the role of exercise in maintaining joint health [60]. Versus Arthritis outline the potential suitability of low impact exercises as appropriate for OA but there is strong emphasis on personal suitability [60]. Therefore, it was established that any PA recommendations do not need strong bias towards a specific type of exercise. In terms of the digital technologies to support the management of KOA, four of the five studies used a web-based platform to deliver their digital intervention whereas only one used mHealth technology.

#### Focus groups

Twenty-six participants were involved in the focus groups. Table 2 presents summaries of the key findings from the thematic analysis along with illustrative quotes from focus groups one and two. Participants identified that they felt PA was important to help manage their OA and overall wellbeing. Individuals also identified that a lack of information led to frustration, whilst the importance of social support was recognised by many. In relation to technology, participants were open to using it and felt that there was a benefit to be able to track PA and symptoms.

**Table 2:** Key findings and illustrative quotes from the focus groups

Key Finding	Participant Quotes
PA important for OA and overall wellbeing	*“These is hope for people who feel it is a sudden onset, you can improve your condition” (Focus group, female participant)* *“For me, physical activity is necessary (being outside, walking around, that kind of thing) for a general sense of wellbeing. If I’m stuck indoors for two long, I get lethargic, get a headache, it’s really unpleasant” (Focus group, male participant)*
A lack of information and knowledge leading to frustration	*“I don’t want week by week or month by month support. It’s a lack of knowledge that’s a problem” (Focus group, female participant)* *“It would be lovely if you could log on to the internet and just get your course of action for the next five years” (Focus group, female participant)* *- “I don’t feel like I am managing it because I don’t feel in control. I don’t know what to do” (Focus group, female participant)*
The importance of social support	*“Many of us here, feel like we are on our own trying to figure things out” (Focus group, female participant)* *“Exercises are boring, there is no question, unless you do them in a group” (Focus group, female participant)*
Real benefits in being able to track PA and symptoms	*“I think over a period of time, it’s useful because if you’re feeling fed up, and you actually look back and you can actually see ‘well I’m a bit better than I was’, that’s helpful” (Focus group, female participant)* *“It’s not a mechanism for making yourself better, but it’s a mechanism for seeing it in context of ‘it’s not always bad”…it’s motivating” (Focus group, female participant)* *- “I like to see that I am not sitting doing nothing all day – I have done a certain amount of paces, because I can’t go and walk for miles and miles with the dog, so I like to keep active and see what I have done” (Focus group, female participant*
Use of technology	*“We are in the generation that are trying to keep up with their children, so if technology helps with that then great” (Focus group, female participant)* *“I haven’t seen the value of it before, but if somebody can convince me of the value, I would give it a go” (Focus group, female participant)*

#### Individual interviews

Five qualified physiotherapists agreed to take part in the individual interviews which lasted on average 81 min (range 66–96 min). Table 3 presents summaries of the key findings from the thematic analysis along with illustrative quotes for the individual physiotherapist interviews. The physiotherapists reported that assessing the suitability of exercises, modifying activities and working within pain limits to suit the individual were critical factors when supporting someone with KOA.

**Table 3:** Key findings and illustrative quotes from physiotherapist interviews

Key Finding	Physiotherapist Quotes
Responding to pain	*“If you can get pain management sorted and you can get sleep, …, then, everything else is much easier. So often those are the starting points to enable self-management”(P3, female)* *“I try and get away from thoughts of ‘pain equals damage’ or ‘if you feel pain you must stop’ because that’s not a good message. If your knee is gently saying, ‘I’m here and its generally tolerable or it’s aching a little then that’s fine’, carry on and see how it goes” (P4, female)* *“Usually, it’s not a ‘you have to stop doing this’, it’s a ‘you can find a way around it’ and trying to think of the whole spectrum of the condition and how it could be adapted to suit the patient” (P4, female)*
Modifying activities seen as key to remain active in enjoyable and sociable activities	*“I think pacing is quite important…so if they gardened all day and then, that night, they had disrupted sleep because of their overactivity, I would then go back to talking about how important it is that they introduce an activity and build up their levels of activity” (P3, female)* *“I would say if somebody’s hobby has always been gardening, we need to ask how we can modify this with aids, or, for example reducing kneeling time, changing sitting positions *etc.*” (P4, female)* *“Some activities like golf for example are very social, so it important to try and understand how we can keep them involved with these activities due to the other benefits, but modifying it to better suit them, such as using a buggy or an electric caddy”(P2, female)*
Assessing the suitability of exercises	*“So, I’d probably ask, initially, their impression of the class and how they felt. Did they come out feeling confident, did it challenge them, did they feel happy coming out of the class, so actually more of their emotional side of how they felt and then I’d look at questioning them really about how it was then affecting their, knees and the pain they are experiencing” (P5, male)* *“So straight away, on the cardio list, I would say, generally, the ones that I would definitely say are ‘go-to’s’ are the swimming, the cross trainer and the Nordic walking, because they’re generally around the sort of low impact and you’ve got support as in the water” (P4, female)*

#### Intervention guiding principles

Data from the focus groups, qualitative interviews and the evidence and theory were used to create the intervention guiding principles which are presented in Table 4. These contain both intervention design objectives and key features of the intervention which were modified throughout the planning and optimisation phases. As an example, participants identified that the app should include rehabilitation exercises that the users could watch whenever needed (design objective). To support this, the key features needed were identified as (i) a bank of rehabilitation exercises, (ii) videos of appropriate techniques and instructions and (iii) resources for monitoring project progress such as an activity timer.

**Table 4:** Guiding principles of the iKOALA intervention

Intervention design objectives	Key features
Access to detailed information relating to OA and the benefits of physical activity	• Disease education materials • Information about the benefits of physical activity • Specific information about a range of physical activities
Ability to recommend appropriate physical activities	• Physical activity recommendations based upon defined user preferences including on how to adapt activities
Ability to deliver physical activity feedback	• Physical activity feedback graphs • Display several different outcomes (i.e., steps, distance) • Physical activity goal setting feature
Ability to record weight and set targets	• Weight recording feature • Target setting feature
Access to targeted rehabilitation exercises	• Bank of rehabilitation exercises available • Videos of appropriate techniques and instructions • Resources for monitoring progress (i.e., activity timer)
Intelligently designed interface to enable personalisation	• Flexible design – individuals can use some elements without having to engage with others • Ability to disable features within settings
Integrate in app opportunities for social support	• Inclusion of social forums • Sharing of success stories • Possibility to “buddy up” using forum features
Allow grading of goals and reward structures	• Activity planning broken into subtasks • In-built reward aspects • Use of positive language throughout
Build app for sustained use	• In-built news “feed” • Notifications relating to reward structures • “Open” structure for long term use
Health status recording	• Ability to record symptoms at any time • Responsive measures related to completion of activities

These guiding principles were used to develop the first iteration of the iKOALA intervention which were incorporated four key focal areas:

- Providing detailed information relating to OA: To overcome the potential lack of education as a barrier to exercise [9], the iKOALA intervention included a comprehensive information section on knee OA using information obtained from the charity Versus Arthritis [60]. This included information on topics such as what is KOA?, how will KOA affect me?, exercise for KOA, weight management, reducing the strain on your knees, coping with low mood and sleep, pharmacological drugs (and other pain relief) for KOA as well as information on surgery.
- Personalised PA recommendations: Feelings of embarrassment and distress at undertaking unsuitable activities and inappropriate PA intensities have been identified as barriers to sustained exercise [9]. Because of this iKOALA was designed to provide personalised PA recommendations. After completing the iKOALA questionnaire to assess their PA preferences (mode and intensity of exercise), users were recommended a range of activities that were specifically suited to their preferences. This allowed users to select only exercises that were suited to them to safely meet their PA goals.
- Self-management support: Behaviour change techniques that have been shown to help with increases in PA behaviour [61, 62], namely goal setting, provision of personal biofeedback, and the prompting of self-monitoring. Additionally, PA tracker integration enabled daily step goals to be displayed in iKOALA with traffic light colours providing visual feedback on whether users were meeting their PA targets (green), almost meeting them (amber) or not close to (red), as well as the use of PA graphs and motivational messages promoting engagement [63].
- Providing opportunities for social support: Because social support has been shown to be advantageous for supporting OA PA programmes [9, 36], the iKOALA included functions to facilitate social support including the identification of local exercise facilities and clubs and an in-app activity specific group chat function where users could post about their experiences and chat with other users.

### Intervention optimisation

#### User trial

Seven participants undertook a 3-week user trial of the iKOALA intervention. All participants completed the user diary and evaluation interview following completion of the trial which lasted 70 min on average (range 62–73 min). Participants gave both positive and negative feedback regarding the iKOALA intervention which following a thematic analysis allowed for key issues to be identified. The feedback and subsequent changes that were agreed to be implemented are presented in Table 5 which included changing the layout of the pages within the app to make the user experience more intuitive and to ensure that only relevant information was shown to the users where personal preferences were applied.

**Table 5:** Changes made to the iKOALA intervention based on patient feedback

Study phase	Feedback	Changes made to iKOALA
Think-aloud	When adding activities to ‘My activity plan’ several participants missed that there were 2 separate tabs for ‘Cardio’ and ‘Mind and body’ activities	We agreed to change the layout of this page so that all activities (including Cardio and Mind and body) we viewable on the same scrollable page but separated out into their respective sections rather than using tabs
Think-aloud	Many participants were unclear on how to access information about a particular activity by pressing on the image associated with the activity	We agreed to add a ‘More info’ button under the image to clarify how to access this information
Think-aloud	Several participants were unclear on how to ‘add a completed activity’ to their exercise plan	We agreed to add the text ‘Completed this week?’ text to the image to clearly identify where to add an activity
Think-aloud	Several participants could not determine how many activities had been completed in the ‘My activity plan’	We agreed to add a green tick when the task had been completed along with the number of times it had been performed (e.g., X1 for once and X2 for twice and so on
User trial	Several participants identified that steps and other activity data recorded through the Fitbit were not synced with the iKOALA app causing confusion	We agreed to make changes to the way the iKOALA app requested information from Fitbit to try to speed up the syncing process
User trial	Several participants were confused at ‘Opportunities in their local area” being displayed in text and on a map in the same page	We agreed to simplify this by just showing local opportunities on the map
User trial	Several participants felt that some activities were not relevant to them following the PA questionnaire	We agreed to review the suitability of the exercise to ensure they were relevant to individuals based on answers given in the PA questionnaire

All participants also completed the system usability scale and reported mixed responses to the usability of the iKOALA intervention (Table 6). Whilst the most common response from participants indicated that the app was felt to be complex, cumbersome, and not necessarily easy to use, most participants also reported that they would like to use the app frequently.

**Table 6:** System usability scores

Question	Modal value	Mean (SD)
I think that I would like to use this app frequently	4	3 (1)
I found this app unnecessarily complex	4	3 (1)
I thought this app was easy to use	3	3 (0)
I think that I would need assistance to be able to use this app	4	4 (1)
I found the various functions in this app were well integrated	3	3 (1)
I thought there was too much inconsistency in this app	4	4 (0)
I would imagine that most people would learn to use this app very quickly	3	3 (1)
I found this app very cumbersome/awkward to use	4	3 (0)
I felt very confident using this app	3	3 (0)
I needed to learn a lot of things before I could get going with this app	3	3 (1)

1 = strongly disagree, 2 = disagree, 3 = neither agree nor disagree, 4 = agree, 5 = strongly agree

#### Think aloud sessions

Six participants took part in one think aloud session which lasted an average of 62 min (range 54–76 min). The overall impressions with the intervention were positive with participants also identifying areas of the app that needed further work to ensure it was clear and logical to the user. Table 5 summarises the key issues identified during the think-aloud sessions and the changes that were implemented. Examples of the overall impressions of the iKOALA intervention were as follows:

*“This does have things that are relevant to your condition, so that’s what sets it apart from the others.... the recording of your pain, the information that you had so you can easily access that”(P5, male)*

*“I really liked the sort of things it records. I like the fact that it’s got the health and the activity summaries, and I thought the health summary was really good….it does make it more specific to a certain type of problem” (P2, female)*

*“I thought it was good, it would certainly motivate you to do something about your condition I think” (P6, female)*

**Fig. 3:**
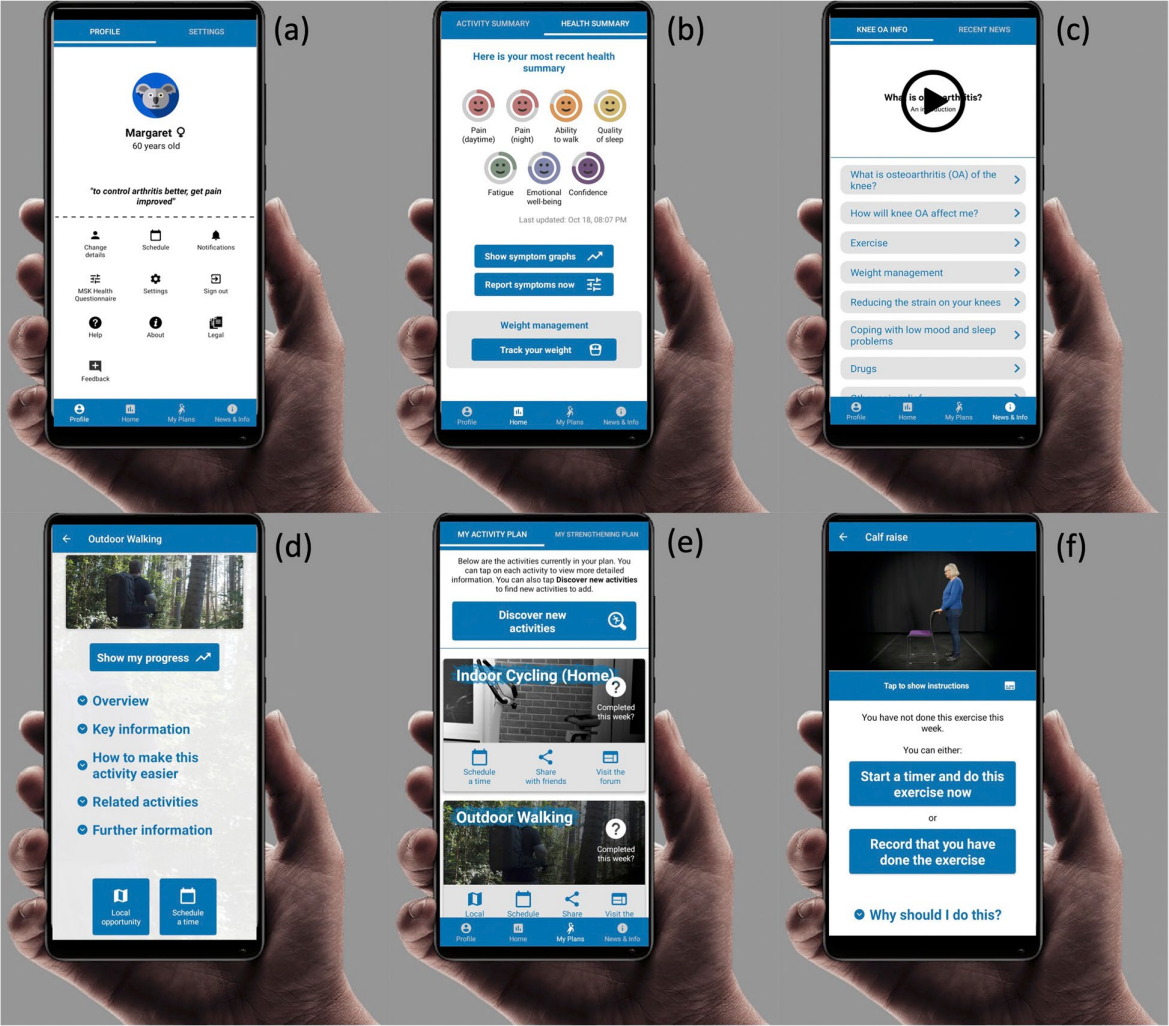
Screenshots of the features and final design of the iKOALA intervention

Images depicting the features and final design of the iKOALA intervention are displayed in Fig. 3 showing (a) the profile page, (b) the health summary, (c) information page, (d) information on outdoor walking, (e) users activity plan, and (f) a calf raise exercise.

## Discussion

This paper outlines the development process of a novel mHealth app for the self-management of KOA using the PBA framework [32]. The use of this person-centred approach for this intervention builds on previous studies that have successfully developed eHealth interventions for a range of health issues including promoting PA [64], reducing cognitive decline [65] and self-managing hypertension [66] by combining behaviour change theory with patient involvement. The development of a mHealth app to support PA in KOA patients is needed, not only because of the beneficial effect that PA has been shown to have on KOA [4–6]. It has also been established that beyond short-term care, the lack of information and (social and professional) support have been identified as barriers to PA, which often lead to a decline in PA leading to poorer health outcomes [7, 9].

Whilst over twenty mHealth apps have been identified in a recent systematic review to support people with KOA [23], these technologies focused on the development of OA assessment, measurement and motion monitoring tools. A small number of other studies have identified improvement in exercise adherence [25] and mobility [24] from mHealth technologies for OA lifestyle management, however, there is some evidence to suggest that the long-term maintenance of PA in knee KOA patients requires a combination of personalised PA guidance, education, and social support. As such there is an unmet need for a comprehensive mHealth application to extend the support for PA and musculoskeletal health in individuals with KOA.

In this study, the planning phase consisted of collating information from the literature along with feedback obtained from focus groups with potential users and interviews with physiotherapists. By triangulating these sources of data, we were able to gain a comprehensive understanding of the elements needed for a self-management tool and subsequently to identify clear guiding principles for the development of the iKOALA intervention. By seeking the views of physiotherapists working with KOA patients, we broadened the perspective of those with experience in this field, enhancing the feedback provided to the development team which may be a useful approach to those developing other eHealth or mHealth interventions.

Key themes that arose from the planning phase identified that people with KOA recognised the importance of PA for their overall wellbeing. However, a lack of social support and a lack of information/knowledge on how best to manage their condition were identified as barriers to engaging in PA which is consistent with other reports [9, 67]. In relation to technology use, despite the cost wearable technology being a concern to some, there was generally a positive attitude to the use of technology to help them self-manage their condition. This is also seen in other groups of older adults [68] including those with multiple long-term conditions [69].

In the optimisation phase, the short-term user trial provided detailed feedback on the intervention, including what worked well, as well as those aspects that were not clear or did not work as intended, and this is similar to other studies adopting this approach [70]. This feedback gave clear direction to the development team on how to improve subsequent iterations of the iKOALA intervention. These changes were then checked with potential users during the think-aloud sessions to ensure they worked as intended. In the final part of the think-aloud sessions, users reported being impressed by the amount of progress made by the development team and were confident it would be useful for supporting them in self-managing KOA.

There are, however, several challenges that remain to be resolved. Whilst most of the issues identified within the table of changes process based on user feedback were rectified, several ‘would like to do’s’ were unbale to be actioned due to the development costs associated with the modifications. These were related to being able to personalise an ‘activity’ within the library of exercises and further personalisation of how the real-time PA data was displayed. There were also instances where inconsistent feedback from users did not provide a clear direction to the development team as to the most appropriate action to take (to make changes or not). As such further feedback is necessary from a broader range of users to clarify any action required. This is hoped to be addressed during the longer user trial.

The main strength of this study was that it followed a structured best-practise methodology and agile iterative development and optimisation process [32]. Whist this process has not been utilised to develop mHealth applications for OA previously, it has been successfully used to develop other mHealth applications. A potential limitation of this study may be the smaller number of participants in the optimization phase compared to the development phase. A greater number of participants may have provided richer information during the user trial and think-aloud sessions. Unfortunately, the recruitment of volunteers for these studies was particularly challenging due to the ongoing COVID-19 pandemic. The next steps of the intervention will be to assess the most recent version of the iKOALA intervention in a user trial of around 12 weeks. During this trial we will seek to monitor PA data, app usage data as well assessing the effect of the trial on musculoskeletal symptoms. Following this, a randomised controlled trial in collaboration with a physiotherapy provider would allow us to investigate the effectiveness of the iKOALA intervention in a real world setting with minimal bias or confounding factors.

## Conclusions

Using a systematic person-based development approach, we have developed the iKOALA to help people with KOA maintain an active lifestyle. Further user testing will refine the intervention to ensure its appropriateness before validating it in a real work setting.

### Supplementary Information

**Supplementary material 1.**

## Data Availability

The datasets generated and/or analysed during the current study are not publicly available but are available from the corresponding author on reasonable request.

## Abbreviations

eHealth: Electronic health
iKOALA: Intelligent knee osteoarthritis lifestyle app
KOA: Knee osteoarthritis
mHealth: Mobile health
NHS: National Health Service
OA: Osteoarthritis
PA: Physical activity
PBA: Person-based approach
REACH: Department for Health Research Ethics Committee
SDT: Self-determination theory
SUS: System usability scale
