# Supplementary material for: Development of the intelligent knee osteoarthritis lifestyle app: a person-based approach
Source: BMC Musculoskelet Disord. 2024 Mar 2;25:189. doi: 10.1186/s12891-024-07313-4 (PMC10908108; doi:10.1186/s12891-024-07313-4)
Supplement: Supplementary file 1 — Supplementary material 1. [file 12891_2024_7313_MOESM1_ESM.docx]

GRIPP2 reporting checklist (short form)

| **Section and**  **topic** | **Item** | **Reported on page no.** |
| --- | --- | --- |
| 1: Aim | Report the aim of PPI in the study | The aim of the present study was to develop and optimise a novel digital app-based intervention for the self-management of KOA with target users. Accordingly, this paper describes the development process of a theory-, evidence- and person-based mHealth app to extend the support for PA and musculoskeletal health beyond short-term, structured rehabilitation through personalised PA, self-management, education, and social support. |
| 2: Methods | Provide a clear description of the methods used for PPI in the study | The development of the intelligent Knee Osteo-Arthritis Lifestyle App (iKOALA) intervention involved an interconnected 2-stage process comprising intervention ‘planning’ and ‘optimisation’ which was informed by the person-based approach (PBA) framework for the development of digital health interventions. The ‘planning’ phase of the PBA involved collecting data from a range of sources including a literature review and primary qualitative data obtained from focus groups with individuals with KOA and interviews with physiotherapists to generate ‘guiding principles’ for the intervention. The ‘optimisation’ phase involved usability testing and qualitative ‘think aloud’ sessions with potential beneficiaries to refine the development of the intervention. |
| 3: Study results | Outcomes—Report the results of PPI in the study, including both positive and negative outcomes | PPI contributed to the study in several ways:   - Four focus group with 26 participants were conducted with individuals diagnosed with KOA to gain a deeper understanding of the impact of living with OA and the needs of those wishing to maintain safe and appropriate exercise and physical activity. - Five qualified physiotherapists agreed to take part in the one-to-one interviews to gain a better understanding of how physiotherapists approached promoting, recommending, and adapting exercises and physical activities for patients with KOA. - A 3-week user trial of the iKOALA intervention was conducted with 7 participants with the aim of gaining feedback on the most up-to-date version of the iKOALA intervention. - Think-aloud sessions were conducted with 6 participants to gain the views of participants about the usability and intuitiveness of the app |
| 4: Discussion and conclusions | Outcomes—Comment on the extent to which PPI influenced the study overall. Describe positive and negative effects | Patient and public involvement in this study was effective in the co-creation of this intervention and led to the development of clear guiding principles for the key features of the application. During the think-aloud sessions The overall impressions with the intervention were positive with participants also identifying areas of the app that needed further work to ensure it was clear and logical to the user. Feedback from participants on the system usability scale identified that whilst the most common response from participants indicated that the app was felt to be complex, cumbersome, and not necessarily easy to use, most participants also reported that they would like to use the app frequently. |
| 5: Reflections/  critical perspective | Comment critically on the study, reflecting on the things that went well and those that did not, so others can learn from this experience | The main strength of this study was that it followed a structured best-practise methodology and agile iterative development and optimisation process. A potential limitation of this study may be the smaller number of participants in the optimization phase compared to the development phase. A greater number of participants may have provided richer information during the user trial and think-aloud sessions. Unfortunately, the recruitment of volunteers for these studies was particularly challenging due to the ongoing COVID-19 pandemic. |
